# Stimulatory Effect of Aluminum in Root Development of *Pogostemon cablin*: Integration of ROS Homeostasis and Gene Expression Networks

**DOI:** 10.3390/ijms262010056

**Published:** 2025-10-15

**Authors:** Zongyu Deng, Zhongqi Lin, Hulan Yang, Cuiyue Liang, Weizhen Jiang

**Affiliations:** 1 School of Traditional Chinese Medicine, Guangdong Pharmaceutical University, Guangzhou 510006, China; 13487456369@163.com (Z.D.); euphoria1956@outlook.com (Z.L.); 15971578579@163.com (H.Y.); 2 Root Biology Center, State Key Laboratory for Conservation and Utilization of Subtropical Agro-Bioresources, College of Natural Resources and Environment, South China Agricultural University, Guangzhou 510642, China

**Keywords:** aluminum, patchouli, root growth, reactive oxygen species (ROS) homeostasis

## Abstract

On acid soils, aluminum (Al^3+^) is typically toxic to plants, though certain species like *Pogostemon cablin* (patchouli) show growth stimulation. This study reveals that Al functions as a root development stimulant in patchouli under acidic conditions. Treatment with 1.0 mM AlCl_3_ for 34 days significantly enhanced root architecture, increasing total root length by 172.12% and root dry weight by 161.75%, without affecting shoot biomass. Structural analysis showed Al accumulation in root tip meristems and lateral root primordia, triggering a 103.77% increase in meristem activity and a 111.9% promotion of cell elongation. Physiological assays showed that Al treatment reduced H_2_O_2_ and malondialdehyde (MDA) levels by 49.2% and 67.6%, respectively, while boosting glutathione (GSH) content by 187.5%, thereby mitigating oxidative membrane damage mainly through the non-enzymatic antioxidant system. Moreover, Al deprivation impaired lateral root elongation, highlighting its functional importance. Gene expression profiling further indicated that Al regulated pathways related to cell proliferation, cell wall remodeling, and lateral root development. Taken together, our findings uncover a novel mechanism by which Al, traditionally regarded as toxic, acts as a stimulator of root development in patchouli, providing new insights into the molecular networks underlying plant abiotic stress responses.

## 1. Introduction

Aluminum (Al) is the most abundant metallic element in the Earth’s crust, primarily existing in solid forms such as aluminosilicates and alumina in neutral and weakly acidic soils [1]. However, when soil pH drops below 5.5, Al solubilizes into toxic forms Al^3+^ or Al (H_2_O)_6_^3+^ [2,3,4]. These soluble Al species can severely inhibit plant growth by disrupting root development, reducing nutrient and inducing the accumulation of reactive oxygen species (ROS) and cellular damage, ultimately leading to reduced crop yields [1,5,6,7,8]. Among the multiple detrimental effects, root growth inhibition is the most characteristic symptom of Al toxicity [9,10,11]. The root apex is widely regarded as the primary target, where Al accumulates in the meristem, elongation, and transition zones [11,12,13,14,15]. By binding to cell wall and plasma membrane components [16,17,18,19], Al reduces cell wall extensibility, alters membrane fluidity, and disrupts DNA stability, thereby impairing both cell division and elongation [20,21,22,23]. Consequently, Al toxicity in acidic soils remains a major factor limiting crop productivity worldwide.

To mitigate Al toxicity, plants have evolved a range of detoxification strategies, broadly classified into two categories: external and internal mechanisms [8,24,25]. Exclusion mechanisms include the secretion of organic acid anions, phenolic compounds, and mucilage, or rhizosphere alkalization, which together prevent Al^3+^ from entering root tip cells [25,26,27,28,29]. Once Al enters the cytoplasm, internal mechanisms mitigate its toxicity through immobilization in the cell wall, chelation with organic acids or phosphate, vacuolar sequestration, and antioxidant defenses [25,29,30]. Among these, redox regulation is particularly important, since Al stress triggers excessive accumulation of ROS, disrupting redox homeostasis and causing oxidative damage to root tissues [8,23,31,32]. To counter Al-induced oxidative stress, plants have evolved two major antioxidant strategies: enzymatic and non-enzymatic systems [33]. The enzymatic system mitigates oxidative damage by enhancing the activity and expression of key antioxidant enzymes such as superoxide dismutase (SOD), peroxidase (POD), catalase (CAT), glutathione S-transferase (GST), and glutathione reductase (GR) [32,34,35,36]. In *Sorghum bicolor*, ROS accumulation co-localizes with *multidrug and toxic compound extrusion* (*MATE*) gene expression, suggesting that ROS signaling contributes to Al resistance [37]. The non-enzymatic system, involving antioxidants such as glutathione (GSH) and flavonoids, provides an additional layer of defense. In rice, GSH alleviates Al toxicity in the root transition zone by activating the Ascorbate–Glutathione (ASA-GSH) cycle, promoting proline synthesis, reducing cell wall-bound Al, and facilitating vacuolar sequestration via GSH-derived phytochelatins [38]. Similarly, in soybean, *GmSTOP1–3* (*Sensitive to Proton Rhizotoxicity 1-3*) enhances Al tolerance by upregulating flavonoid biosynthesis genes such as *GmCHS* (*Chalcone synthase*) and *GmIFS* (*isoflavone synthase*), leading to flavonoid accumulation and improved ROS scavenging [23].

Beyond its well-established toxicity, Al has also been reported to exert beneficial effects on certain plant species adapted to acidic soils, which are widely distributed in tropical and subtropical regions [8,26,27,28], such as *Melastoma malabatbricum*, *Tabebuia chrysantha*, *Quercus Serrata*, *Symplocos paniculata*, *Camellia japonica*, and *Camellia sinensis* [29,30,39,40,41,42]. For instance, in *M. malabathricum*, exposure to 0.5 mM Al enhances root activity and facilitates nutrient uptake, including phosphorus (P), potassium (K), calcium (Ca), and magnesium (Mg) [29,43]. In *Camellia japonica*, treatment of 2-year-old plants with 0.5–1 mM Al promotes lateral root proliferation and increases photosynthetic efficiency [42]. While in *Camellia sinensis* (tea), treatment with 1.0 mM Al^3+^ is even essential for root growth and development, as evidenced by the failure to generate new roots in Al^3+^-deprived conditions and rapid root tip damage observed within 1 day of Al deprivation. Structural analysis of root tips further suggests that Al^3+^ is indispensable for root meristem development and activity, primarily through its role in maintaining DNA integrity within meristematic cells [44]. These findings reveal a striking duality: Al acts as a toxin in most plants yet serves as a growth-promoting or even essential element in certain acid-adapted species [42,43,44,45]. However, the molecular and physiological mechanisms underlying these beneficial effects remain poorly understood.

*Pogostemon cablin* (Blanco) Benth., a perennial aromatic herb of the Lamiaceae family, is an economically important medicinal and industrial plant. Its essential oil, rich in patchouli alcohol, is widely used in traditional Chinese medicine, as well as in perfumes, cosmetics, and food products [46,47,48,49,50]. Patchouli is native to tropical Southeast Asia and has been cultivated for centuries in southern China, particularly in Guangdong, Guangxi, and Hainan provinces [46,51], which are characterized by acidic soils with high Al content [52]. The species thrives in these warm, humid climates and prefers loose, fertile, acidic sandy loam soils [46,53]. Notably, studies have reported that patchouli grown in Guangdong and Hainan accumulates Al at concentrations ranging from 0.91 to 1.74 mg/g [54,55], suggesting that patchouli may act as a potential Al hyperaccumulator with exceptional tolerance to Al toxicity. Nevertheless, the effects of Al on patchouli growth and the mechanisms underlying its tolerance or adaptation remain largely unexplored.

In this study, we demonstrate that Al exerts beneficial effects on root system architecture in patchouli. Al significantly promoted root growth by increasing the density of adventitious and lateral roots, accelerating lateral root primordia formation, and enhancing both meristematic activity and cell elongation. Moreover, Al deprivation experiments further revealed that sustained Al availability is critical for lateral root elongation. These growth-promoting effects were closely associated with modulation of ROS metabolism and the correlated with differential transcript levels for genes involved in cell proliferation, cell wall remodeling, and root development. Collectively, these findings highlight a novel role of Al as a stimulator of root growth in patchouli, providing new insights into the dual functions of Al in plants and offering potential strategies to improve crop resilience in acidic soils.

## 2. Results

### 2.1. Al Promotes Root Growth in Patchouli Seedling

**Figure 1:**
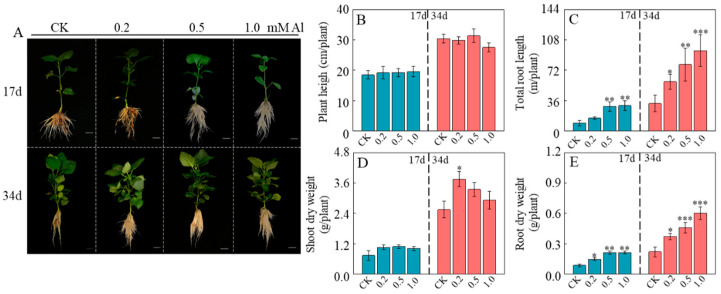
Effect of Al treatment on growth of patchouli seedling. (**A**) Phenotype of patchouli treated with different Al concentrations at pH 4.5 after 17 and 34 days of cultivation; (**B**) Plant heigh; (**C**) Total root length; (**D**) Shoot dry weight; (**E**) Root dry weight. Bar = 1 cm. Asterisks indicate significant differences between Al treatments and the control in the Student’s *t*-test (* *p* < 0.05; ** 0.001 < *p* < 0.01; *** *p* < 0.001).

Al significantly promoted root growth in patchouli seedlings (Figure 1A). Compared to the control (0 μmol/L AlCl_3_), seedlings treated with 0.2, 0.5 and 1.0 mM Al for 17 days showed increases in total root length by 60.97%, 211.58%, and 222.05%, respectively (Figure 1C). This growth-promoting effect was sustained over time, with increases of 64.71%, 140.6%, and 172.12% observed after 34 days of treatment (Figure 1C). Similarly, root dry weight showed a consistent response, exhibiting significant increases of 55.12%, 112.44%, and 161.75% under corresponding Al concentrations after 34 days of treatment (Figure 1E). These findings provide strong evidence that Al can significantly promote patchouli root growth. Interestingly, this stimulatory effect was absent in shoots (Figure 1A), as plant height and shoot dry weight showed no significant differences compared with the control (Figure 1B,D). These findings clearly demonstrate that Al selectively stimulates root development without affecting shoots growth.

### 2.2. Al Enhances Cellular Activity in Root Meristematic Zones to Stimulate Root Growth

**Figure 2:**
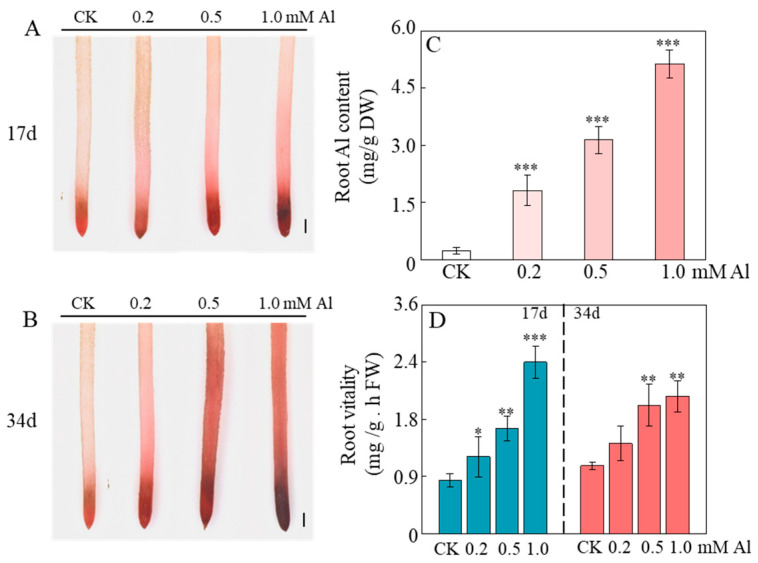
Effect of Al treatment on root activity of patchouli seedling. (**A**,**B**) were 2,3,5-triphenyltetrazolium chloride staining in lateral root of patchouli seedlings treated with different Al concentrations at pH 4.5 after 17 days and 34 days, respectively; (**C**) Root Al content after 34 days of exposure to different Al concentrations; (**D**) Root vitality. Bar = 2 mm. Asterisks indicate significant differences between Al treatments and the control in the Student’s *t*-test (* *p* < 0.05; ** 0.001 < *p* < 0.01; *** *p* < 0.001).

To determine the distribution of Al in the roots, in situ morin staining was performed. The results revealed that Al was predominantly accumulated in regions with high cellular division activity, such as the root tip meristematic zone and lateral root primordia (Figure S1C,D), as evidenced by a significantly stronger fluorescence signal in the meristematic zone compared to the elongation zone (Figure S1A,B), suggesting that Al may promote root growth by specifically modulating cellular activity in the root apical region. To validate this hypothesis, root activity was quantitatively evaluated using TTC staining. The results demonstrated a pronounced increase in root tip staining intensity with higher Al concentrations, particularly during the early treatment stage (Figure 2A,B). Quantitative analysis indicated that exposure to 1.0 mM Al for 17 days enhanced root activity by 224.09% compared to the 0 mM AlCl_3_ control (Figure 2D), while a 103.77% increase was observed after 34 days of treatment (Figure 2D). These results support the conclusion that aluminum stimulates root growth by enhancing cellular activity in meristematic tissues of the root tip.

### 2.3. Al Enhances Cell Elongation in the Root Elongation Zone to Promote Root Growth

**Figure 3:**
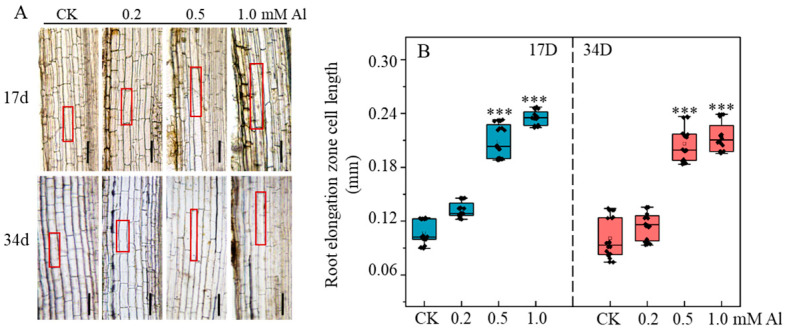
Effect of Al treatment on cell length of root elongation zone of patchouli seedling. (**A**) Photographs of longitudinal sections of the root elongation zone (Bar = 100 μm); (**B**) Cell length of root elongation zone. The red square outlines the elongation zone cells. Data represent mean ± SD of n = 20 individual root cortical cells measured from at least 5 independent plants. Asterisks indicate significant differences between Al treatments and the control in the Student’s *t*-test (*** *p* < 0.001).

Al not only enhances root activity but also promotes cell elongation in the root elongation zone (Figure 3A). To examine this effect, we measured cell length in the elongation zone of patchouli roots treated with varying Al concentrations for 17 and 34 days. The results showed that Al treatment significantly increased cell length in the root elongation zone of patchouli. After 17 days, 0.2 mM Al increased cell length by 24.5% compared to the 0 mM AlCl_3_ control (Figure 3B). This effect was further amplified at higher concentrations, with cell length increasing by 97.1% and 104.3% under 0.5 mM and 1.0 mM Al treatments, respectively (Figure 3B). Moreover, the promotive effect remained sustained after 34 days of exposure (Figure 3B). These results indicate that aluminum specifically enhances cell elongation in the root elongation zone, thereby contributing effectively to overall root growth in patchouli.

### 2.4. Al Treatment Reduces ROS Accumulation in Patchouli Roots

**Figure 4:**
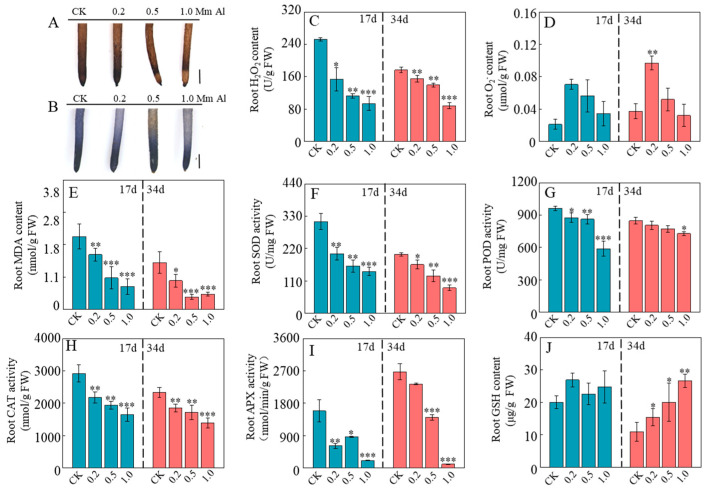
Effects of Al treatment on ROS accumulation and antioxidant enzyme activity in Patchouli Seedling roots. (**A**) 3,3′-Diaminobenzidine (DAB) staining; (**B**) Nitroblue tetrazolium (NBT) staining; (**C**) H_2_O_2_ content in roots; (**D**) O_2_^−^ content in roots; (**E**) MDA content in roots; (**F**) SOD activity in roots; (**G**) POD activity in roots; (**H**) CAT activity in roots; (**I**) APX activity in roots; (**J**) GSH content in roots. Bar = 2 mm. Asterisks indicate significant differences between Al treatments and the control in the Student’s *t*-test (* *p* < 0.05; ** 0.001 < *p* < 0.01; *** *p* < 0.001).

Al treatment markedly reduced ROS accumulation in patchouli roots, as evidenced by a significant decrease in H_2_O_2_ content with increasing Al concentration (Figure 4A,C). Compared with the 0 mM AlCl_3_ control, seedlings exposed to 1.0 mM Al for 17 and 34 days showed reductions in H_2_O_2_ content of 62.6% and 49.2%, respectively (Figure 4C). Similarly, malondialdehyde (MDA) content decreased by 68.96% and 67.6% under the same conditions (Figure 4E), indicating that Al treatment effectively alleviates oxidative damage to root cell. In contrast, O_2_^−^ content increased significantly under 0.2 mM Al but showed no significant changes under higher concentrations (0.5–1.0 mM) (Figure 4B,D), suggesting that low Al levels may transiently stimulate superoxide production, whereas higher levels help stabilize O_2_^−^ accumulation. Moreover, the activities of SOD, POD, CAT, and ascorbate peroxidase (APX) decreased markedly with rising Al levels, showing reductions of 56.65%, 13.9%, 40.16%, and 90.3%, respectively, after 34 days of 1.0 mM Al treatment (Figure 4F–I). Conversely, GSH content rose substantially with rising Al concentration, by 110.3%, 136.15%, and 187.5% under 0.2, 0.5, and 1.0 mM Al, respectively, after 34 days, although no significant changes were observed at 17 days (Figure 4J). These findings suggest that Al alleviates oxidative stress in patchouli roots primarily by enhancing non-enzymatic antioxidant capacity (e.g., GSH), while suppressing the enzymatic antioxidant system (e.g., SOD, POD, CAT, APX).

### 2.5. Al Remodels Patchouli Root Architecture by Boosting Root Initiation and Accelerating Lateral Root Development

**Figure 5:**
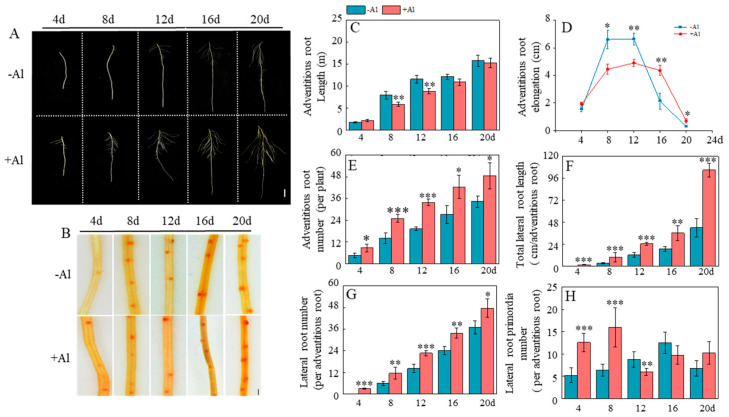
Effects of Al stress on root growth of patchouli seedling. (**A**) Phenotype of adventitious root growth, bar = 0.5 cm; (**B**) 2,3,5-triphenyltetrazolium chloride staining in lateral root primordia, bar = 1 mm; (**C**) Adventitious root length; (**D**) Adventitious root elongation; (**E**) The number of adventitious roots per plant; (**F**) Total lateral root length per adventitious root; (**G**) The number of lateral roots per adventitious root; (**H**) The number of lateral root primordia per adventitious root. Asterisks indicate significant differences between Al treatments and the control in the Student’s *t*-test (* *p* < 0.05; ** 0.001 < *p* < 0.01; *** *p* < 0.001).

To further investigate the effects of Al on the root architecture of patchouli, we monitored the growth dynamics of adventitious and lateral roots. The results revealed that Al exerted a dual promotive effect on lateral root formation by both increasing the number of lateral roots and accelerating the development of lateral root primordia. Specifically, Al treatment significantly enhanced the number and total length of lateral roots, whereas no lateral roots were observed in the control by day 4 (Figure 5A,F,G). From day 4 to day 8, the number of lateral root primordia under Al treatment remained consistently higher than in the control, although this difference disappeared after day 8 (Figure 5B,H), indicating that Al plays a critical role during the early developmental stages of lateral roots. Additionally, Al also significantly increased the number of adventitious roots (Figure 5E). However, from day 8 to day 12, the length of adventitious roots under Al treatment was shorter than in the control, with this difference becoming non-significant after day 12 (Figure 5C). A similar trend was observed in the elongation of adventitious roots (Figure 5D). Collectively, these findings suggest that Al effectively modulates patchouli root architecture by increasing the number of lateral and adventitious roots while specifically promoting the early development of lateral root primordia.

### 2.6. Al Exhibits Non-Essential but Beneficial Effects on Patchouli Root Growth

**Figure 6:**
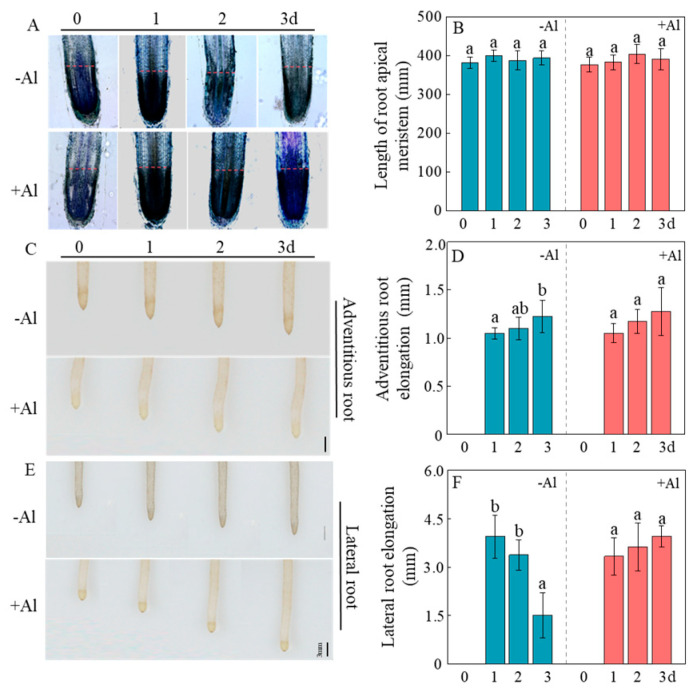
Effects of 3 d +/−Al treatment on root development and elongation after growth on +Al solution for 30 d. (**A**) Photographs of root tip longitudinal sections. The blue color indicates the fluorescence signal of Calcofluor-white bound to root cell walls. The red dashed line indicates the boundary between the root apical meristem and the elongation zone, bar = 100 μm; (**B**) Length of root apical meristem; (**C**) Photographs of adventitious root phenotypes; (**D**) Adventitious root elongation; (**E**) Photographs of lateral root phenotypes, bar = 3 mm; (**F**) Lateral root elongation. Duncan multiple comparison was adopted to analyze the significance of data differences. The columnar chart marked with different lowercase letters demonstrates significant differences between data (*p* < 0.05).

To determine whether Al is essential or beneficial for patchouli root development, seedlings were pre-cultured for 30 days in a nutrient solution containing 1.0 mM AlCl_3_ (pH 4.5) to stimulate the growth of new, healthy white roots. They were then transferred to nutrient solutions containing either 0 or 1.0 mM AlCl_3_ for an additional 3 days. The results showed that the length of the root meristematic zone remained consistent over time, regardless of Al presence (Figure 6A,B and Figure S4A). Likewise, adventitious root elongation also showed no significant change under both Al conditions (Figure 6C,D and Figure S4B). In contrast, lateral root elongation declined markedly in the absence of Al, with elongation on day 3 reduced to only 38% of that observed on day 1 (Figure 6E,F and Figure S4B). This decline was not observed when Al was present, as lateral root elongation remained stable over the same period (Figure 6E,F). These results suggest that although Al is not essential for all types of root growth in patchouli, it is critically involved in sustaining lateral root elongation.

### 2.7. Al Modulates Gene Networks to Enhance Root Growth via ROS Regulation, Cell Proliferation, and Cell Wall Remodeling

**Figure 7:**
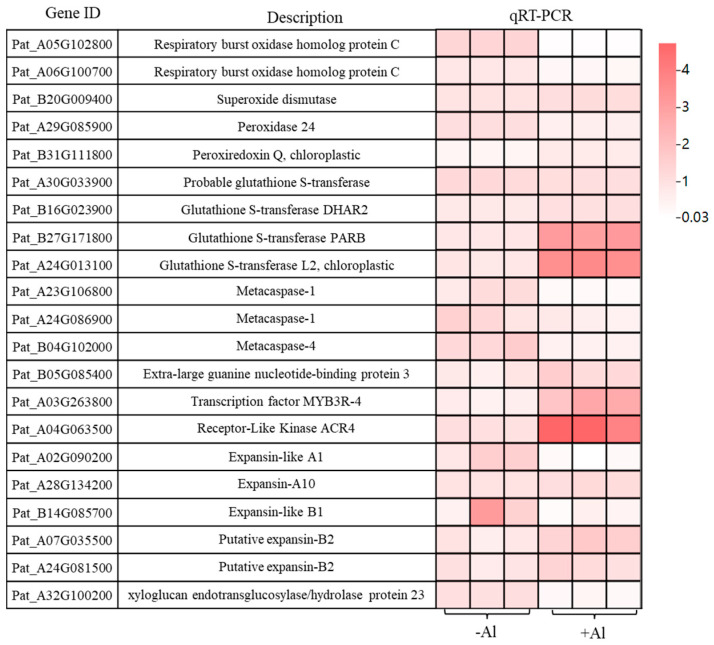
Aluminum-induced expression patterns of genes involved in cell division, ROS signaling, and cell wall modification in patchouli roots. qRT-PCR was conducted to analyze gene expression in roots of patchouli seedlings subjected to 0 or 1.0 mM Al^3+^ treatments. Rows represent individual samples, columns represent genes. Color scale reflects relative expression levels (red: high; white: low).

To further elucidate the molecular mechanisms underlying Al-induced root development in patchouli, we analyzed the expression of genes associated with cell division, antioxidant defense, and cell wall modification using qRT-PCR. Seedlings were cultured for 30 days in a nutrient solution containing either 0 or 1.0 mM AlCl_3_ (pH 4.5). The results revealed that the expression of two *Respiratory burst oxidase homolog protein C* (*RBOHC*) genes was significantly downregulated under Al treatment compared to the 0 mM control (Figure 7), indicating a reduction in ROS production during Al exposure. In contrast, genes encoding *Glutathione S-transferase L2* (*GSTL2*), *Glutathione S-transferase PARB* (*GSTPARB*), and *Peroxiredoxin Q* (*PRXQ*) were significantly upregulated, whereas *Peroxidase 24* (*PER24*) was downregulated. No significant differences were detected in the expression of *Superoxide dismutase* (*SOD*), *Glutathione S-transferase DHAR2*, and probable *glutathione S-transferase* (Figure 7). This transcriptional adjustment suggests that Al fosters a more reducing cellular environment, mitigating oxidative stress. Additionally, the expression of three *metacaspases* (*AMCs*), which are associated with programmed cell death, were significantly downregulated (Figure 7). Conversely, genes related to cell proliferation, such as *Extra-large guanine nucleotide-binding protein 3* (*XLG3*), transcription factor *MYB3R-4*, and *Receptor-like kinase ACR4*, were markedly upregulated (Figure 7), indicating that Al suppresses root cell death while enhancing cell proliferation and meristematic activity, thereby promoting root growth and development. Regarding cell wall modification, the expression of *Expansin-A10* (*EXPA10*) and *two putative Expansin-B2* (*EXPB2*) genes were slightly upregulated, while *Expansin-like A1* (*EXLA1*), *Expansin-like B1* (*EXLB1*), and *xyloglucan endotransglucosylase*/*hydrolase protein 23* (*XTH23*) were significantly downregulated (Figure 7). These results suggest that Al promotes root elongation by selectively activating specific cell wall-loosening genes while repressing other remodeling pathways that might limit cell expansion.

### 2.8. Effect of Al on the Expression of Genes Related to Lateral Root Elongation in Patchoulit

**Figure 8:**
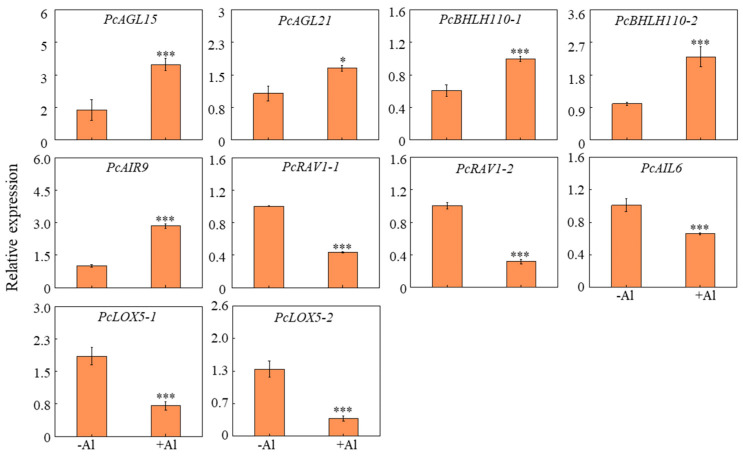
Al modulates the expression of lateral root development-associated genes in patchouli roots. qRT-PCR was conducted to analyze gene expression in roots of patchouli seedlings subjected to 0 or 1.0 mM Al treatments. Data are means of four independent replicates ± SE. Asterisks indicate significant differences between Al treatments and the control in the Student’s *t*-test (* *p* < 0.05; *** *p* < 0.001).

To further investigate the molecular mechanisms underlying Al^3+^-induced effects on lateral root development in patchouli, we analyzed the expression of 10 root growth-related genes using qRT-PCR. The results revealed significant changes in gene expression in response to Al^3+^ treatment. Specifically, the expression levels of *Agamous-like MADS-box proteins* (*AGL15* and *AGL21*), transcription factors *basic helix-loop-helix 110-1 and 110-2* (*bHLH110-1*, *bHLH110-2*), and *microtubule-associated protein* (*AIR9*) were markedly upregulated (Figure 7). Conversely, genes such as two *AP2*/*ERF and B3 domain-containing transcription factor RAV1*, *AP2-like ethylene-responsive transcription factor AIL6*, and two *linoleate 9S-lipoxygenase 5* (*LOX5*) were significantly downregulated in Al^3+^-treated roots (Figure 8). These findings suggest that Al^3+^ may promote lateral root development in patchouli by regulating the expression of specific genes involved in cell division, differentiation, and stress responses.

## 3. Discussion

Aluminum (Al), a non-essential element for plants, is widely known for its phytotoxicity in acidic soils. Its toxicity largely depends on ionic speciation and concentration, with Al^3+^ being the most prevalent and toxic form under strongly acidic conditions (pH = 4.3) [28]. However, a growing body of evidence suggests that Al can also exert neutral or even beneficial effects on certain adapted plant species under specific concentration conditions (e.g., 0.5–1.0 mM Al), challenging the traditional view of its role in plant biology [8,28,42,44,45]. *Pogostemon cablin* (Blanco) Benth, a traditional Chinese medicinal plant widely cultivated in southern China, has been reported to accumulate high concentrations of Al, particularly in regions such as Guangdong and Hainan provinces [54,55]. This study explores the effects of Al on patchouli root growth and development, with a particular focus on root elongation and lateral root formation. Through this investigation, we aim to provide new insights into the dual role of Al as both a potential stressor and a growth enhancer in Al-accumulating plants.

In most plants, Al^3+^ toxicity is characterized by suppressed root growth and distorted root architecture [10,11,19,29]. Upon binding to the cell wall and entering the cells, Al^3+^ reduces cellular flexibility, damages the plasma membrane, disrupts ROS homeostasis, and impairs DNA stability, collectively suppressing cell division and elongation [8,32,56,57]. However, certain plant species adapted to acidic soils exhibit stimulated root growth under appropriate Al^3+^ concentrations [27,42,44]. In this study, we observed a similar phenomenon in patchouli, where Al^3+^ acted not as a toxin but as a specific stimulant for root development. Our results demonstrated that Al^3+^ significantly enhanced root fresh weight and total root length, primarily through promoting the formation of adventitious and lateral roots (Figure 5E,G). Notably, this promotional effect was tissue-specific, as shoot growth remained unaffected (Figure 1 and Figure S3). This distinct response raises the possibility that Al^3+^ influences carbohydrate and nutrient allocation toward the root system, thereby supporting the development of a more extensive absorptive network, although this hypothesis requires further experimental validation. Moreover, this stimulatory effect was strongly dependent on Al^3+^ bioavailability rather than H^+^ activity, as the most substantial improvements in root biomass and architecture were observed under 1.0 mM AlCl_3_ at pH 4.5, a condition favoring high Al^3+^ availability (Figure S2) [25,58]. These findings, consistent with observations in other Al-adapted species like tea plants [47,49], indicate that patchouli not only possesses high Al^3+^ tolerance but also may have evolved an adaptive strategy to utilize Al^3+^ as a stimulator for optimizing root system architecture under acidic conditions.

It is well-established that Al^3+^ inhibits root growth in most plants by strongly suppressing cell division in the meristematic zone, as well as impairing cell expansion and elongation in the transition and elongation zones [25,57,59,60]. Interestingly, in certain Al-accumulating species such as *Camellia sinensis*, Al is indispensable for maintaining meristematic activity and DNA integrity [44]. Similarly, we observed that Al treatment significantly enhanced root tip cell activity in patchouli (Figure 2), accompanied by the upregulation of proliferation-related genes such as *XLG3*, *MYB3R-4*, and *ACR4*, and the downregulation of programmed cell death genes (e.g., *AMCs*) (Figure 7). Furthermore, cell elongation in the elongation zone was also markedly promoted under Al exposure (Figure 3), consistent with the strong upregulation of expansion genes (e.g., *EXPB2*, *EXPA10*) and the downregulation of the *xyloglucan endotransglucosylase*/*hydrolase gene XTH23* (Figure 7). Such transcriptional changes are recognized as critical Al-tolerance mechanisms in plants [61,62,63,64,65], suggesting that Al modulates cell wall remodeling to sustain rapid cell expansion and continuous root growth. Taken together, these findings further support the conclusion that Al functions as a growth stimulator in patchouli, concurrently promoting meristematic cell proliferation, inhibiting premature cell death, and facilitating cell elongation by altering the transcript levels of key genes involved in cell wall modification.

In addition to inhibiting root growth, disruption of oxidative homeostasis is a key driver of Al toxicity in plants. It has been widely reported that Al disrupts ROS balance, leading to oxidative damage in key biomolecules and suppression of root growth [47,49,50,51,52,53]. By contrast, it was found that Al significantly reduced the content of H_2_O_2_ in patchouli roots (Figure 4), indicating that patchouli actively modulates ROS generation and conversion rather than passively suffering oxidative damage. The reduction in MDA content (Figure 4E), which aligns with previous observations in *Camellia japonica* [42], further supports the notion that this ROS modulation reflects a controlled and adaptive response rather than uncontrolled oxidative damage. Unlike H_2_O_2_, O_2_^−^ levels were markedly increased after 34 days of treatment with 0.2 mM AlCl_3_, although they were slightly reduced under 1.0 mM AlCl_3_ compared with the control (Figure 4B,D). Furthermore, the expression of *RBOHC* was significantly downregulated under high Al conditions (Figure 7), suggesting that a feedback inhibition mechanism attenuates *RBOH* activity, thereby preventing excessive O_2_^−^ accumulation and mitigating potential cytotoxicity.

To counteract Al toxicity, plants have evolved a range of adaptive mechanisms, among which the reduction in Al-induced ROS accumulation is particularly critical [23]. In patchouli roots, the activities of major antioxidant enzymes, including SOD, POD, CAT, and APX, were markedly suppressed under Al treatment (Figure 4F–I). These results suggest that the antioxidant enzyme system may not serve as the primary mechanism for ROS scavenging in patchouli under Al stress. Instead, GSH appears to play a pivotal role. As a major non-enzymatic antioxidant, GSH effectively scavenges excess H_2_O_2_, thereby contributing to plant defense against Al stress [66,67]. In rice, GSH has been reported to alleviate Al toxicity by mitigating oxidative stress in the root transition zone (TZ), reducing Al deposition in the cell wall, and enhancing vacuolar Al sequestration in TZ cells [38,68]. Consistently, in patchouli, GSH content increased substantially with rising Al concentrations after 34 days of treatment (Figure 4J), accompanied by the upregulation of *Glutathione S-transferase* genes such as *DHAR2*, *GSTL2*, and *PARB* (Figure 7). These results indicate that GSH functions as a key redox buffer, providing basal protection when enzymatic defenses are weakened and thereby maintaining redox homeostasis. Beyond its antioxidant capacity, GSH also acts as an important developmental signal [69,70]. This implies that in patchouli, GSH may not only mitigate oxidative stress but also act as a signaling mediator that supports root growth under Al stress.

Although the physiological and molecular mechanisms by which Al promotes plant growth have been explored for decades [8,47,48,49], the detailed molecular basis remains poorly understood. In this study, the upregulation of transcription factors such as *AGL15*, *AGL21*, *AIR9*, and *bHLH110* under Al treatment suggests that Al enhances root development by activating pathways regulating cell division, differentiation, elongation, and branching [71,72,73,74], thereby providing a mechanistic explanation for the observed architectural changes in patchouli roots. In contrast, several genes associated with lateral root initiation and development, including *RAV1*, *AIL6*/*PLT3*, and *LOX5*, were significantly downregulated under Al treatment (Figure 8) [75,76,77,78,79], indicating a complex regulatory role of Al in lateral root morphogenesis in patchouli. In particular, the strong repression of *AIL6*/*PLT3*, a key PLETHORA family transcription factor required for developmental zonation and cell division during lateral root initiation [76,77], implies that Al may promote lateral root formation through compensatory mechanisms independent of *PLT3* [14,80,81]. Such mechanisms are likely mediated by hormonal modulation, epigenetic regulation, or alternative transcriptional programs, as further supported by the upregulation of *AIR9* and *bHLH110* [73,74]. Furthermore, the downregulation of *LOX5*, a lipoxygenase involved in lipid metabolism and jasmonic acid signaling, implies that Al may reconfigure hormone signaling pathways, such as ethylene and jasmonic acid, to prioritize root growth over canonical stress responses [75,82]. Similarly, the marked repression of *RAV1*, a negative regulator of growth and stress adaptation [78,79], indicates that Al may alleviate transcriptional repression to promote lateral root initiation and expansion.

## 4. Materials and Methods

### 4.1. Plant Material and Growth Conditions

Patchouli cuttings (8–10 cm in length) were collected from a field site in Foshan, Guangdong, China. The roots were gently rinsed with clean water to remove adhering debris and pre-cultured in deionized water for 3 days to ensure complete removal of residual soil particles. Subsequently, the seedlings were cultivated for 34 days in a nutrient solution (pH 4.5) supplemented with 0, 0.2, 0.5, or 1.0 mM AlCl_3_. The composition of the nutrient solution was as follows: 0.4 mM NH_4_NO_3_, 1.5 mM KNO_3_, 1.2 mM Ca(NO_3_)_2_, 0.125 mM KH_2_PO_4_; 0.3 mM (NH_4_)_2_SO_4_, 0.3 mM K_2_SO_4_, 0.5 mM MgSO_4_, 0.04 mM Fe-Na-EDTA, 0.025 mM MgCl_2_, 0.0015 mM ZnSO_4_, 0.0015 mM MnSO_4_, 0.0005 mM CuSO_4_, 0.0025 mM H_3_BO_3_ and 0.00015 mM (NH_4_)_6_Mo_7_O_24_. The solution was continuously aerated and renewed weekly. After 17 or 34 days of aluminum treatment, plants were harvested and photographed using a digital camera (Nikon D600, Tokyo, Japan). Dry weight and total root length were measured according to the method described by Zhao et al. [83]. Each treatment included four biological replicates.

To investigate the effects of pH and Al^3+^ interactions on the growth of patchouli seedlings, cuttings were first pre-cultured in a nutrient solution for 15 days to facilitate root system renewal. The seedlings were then transferred to fresh nutrient solutions containing either 0 or 1.0 mM AlCl_3_, with pH adjusted to 3.5, 4.5, 5.5, or 6.5, and grown for another 15 days. Throughout the experiment, the nutrient solution was continuously aerated and replaced weekly. Seedlings were photographed and growth parameters were measured following previously described methods. Each treatment was conducted with four biological replicates.

The effect of Al^3+^ on root architecture was evaluated in patchouli seedlings. Cuttings were first pre-cultured in deionized water for 3 days and subsequently grown in a nutrient solution supplemented with 0 or 1.0 mM AlCl_3_ (pH 4.5). Plants were harvested at 4-day intervals throughout a 20-day growth period. At each harvest, plant height and the length of newly formed adventitious roots were measured using a ruler. The number of adventitious roots, lateral roots, and total root length were quantified in accordance with established methods [83]. Each treatment included four biological replicates.

To examine the effects of aluminum withdrawal following initial exposure, seedling cuttings were first cultivated for 30 days in a nutrient solution containing 1.0 mM AlCl_3_ at pH 4.5. The plants were then transferred to a fresh nutrient solution supplemented with either 0 or 1.0 mM AlCl_3_ and grown for an additional 3 days. Adventitious and lateral roots were imaged using a stereo microscope (DM5000B; Leica, Wetzlar, Germany). Root tips were excised for histological analysis using toluidine blue staining and for aluminum localization via in situ morin staining, as detailed in subsequent sections. Root tip elongation was tracked daily over the 4-day period using ImageJ software (v1.43u; National Institutes of Health, Bethesda, MD, USA), and calculated as the difference between final and initial root lengths. Each treatment included four biological replicates.

### 4.2. Detection of ROS Level and Antioxidant Enzyme Activity

Root tips (approximately 1 cm in length) were excised and rinsed with distilled water for 3 min. Subsequently, the samples were immersed in either DAB or NBT staining solution and subjected to vacuum infiltration for 10 min, followed by further incubation in the dye solution for an additional 10 min. After staining, the root tips were rinsed thoroughly with distilled water and imaged under a stereomicroscope (Leica DM5000B, Wetzlar, Germany).

The activities of superoxide dismutase (SOD), peroxidase (POD), and catalase (CAT), as well as the contents of hydrogen peroxide (H_2_O_2_) and malondialdehyde (MDA), were quantified using commercial assay kits (Suzhou Keming Biotechnology Co., Ltd., Suzhou, China) in accordance with the manufacturer’s instructions. Ascorbate peroxidase (APX) activity was determined using a spectrophotometric assay kit (Yisejiu Biotechnology Co., Ltd., Lianyungang, China). The level of reduced glutathione (GSH) was measured with a GSH content assay kit (Solarbio Biotechnology Co., Ltd., Beijing, China). Superoxide anion content was assessed using the CheKine™ Microplate Assay Kit (Abebio Biotechnology Co., Ltd., Wuhan, China), strictly following the provided protocols.

### 4.3. TTC Staining

Newly emerged root tips and adventitious roots were collected at specified time points—root tips after 17 or 34 days of aluminum treatment, and adventitious roots at 4-day intervals over a 20-day growth period. All samples were incubated in 0.01% (*w*/*v*) 2,3,5-triphenyltetrazolium chloride (TTC) solution for 1 h. The reaction was terminated by transferring the roots into 1 mol/L hydrochloric acid for 10 min. Staining was observed under a stereo microscope (DM5000B; Leica, Wetzlar, Germany) using 10% glycerin as transparent reagent.

TTC standard curve establishment: A 0.2 mL aliquot of 0.4% TTC solution was reacted with a suitable amount of Na_2_S_2_O_4_ powder to generate formazan. The mixture was then diluted to 10 mL with ethyl acetate to form a stock solution, which was subsequently serially diluted to obtain standard solutions with concentrations ranging from 25 to 200 μg/mL. Absorbance was measured at 485 nm using ethyl acetate as a blank, and a standard curve was constructed based on the results.

Sample Assay: Root tip samples (0.1 g) were homogenized with an equal volume of 0.4% TTC solution and 2 mL of phosphate buffer (pH 7.4), followed by incubation at 37 °C for 1–3 h in the dark. The reaction was terminated by adding 1 mol/L H_2_SO_4_. The tissue was thoroughly ground, and the homogenate was extracted with ethyl acetate. After bringing the final volume to 10 mL with ethyl acetate, absorbance was measured at 485 nm. The amount of tetrazolium reduced was quantified using the previously established standard curve.

### 4.4. Cell Elongation Measurements

Root tip samples, approximately 2 cm in length, were rinsed with 0.5 mM CaCl_2_ for 3 min and embedded in 7% agar before obtaining longitudinal sections (40 μm) using a microtome (VT 1200; Leica, Wetzlar, Germany). The length of elongation zone cells was imaged using an inverted microscope and quantified with ImageJ software (https://imagej.net/, accessed on 15 May 2024).

### 4.5. Root Structure Analysis

Root tip samples were carefully excised and embedded in 7% agar for longitudinal sectioning into 40 μm slices using a microtome (VT1200; Leica, Wetzlar, Germany). The sections were stained with 0.2% toluidine blue for 1 min and rinsed thoroughly in clean water for 1 min. Stained sections were then examined under a microscope (DM5000B; Leica, Wetzlar, Germany) to observe structural details. The apical meristem length (the distance between the quiescent center and the starting point of the elongation zone) was accurately measured using ImageJ software (1.43u; National Institutes of Health, Bethesda, MD, USA).

### 4.6. Morin Staining

Root tip and lateral root primordia samples were collected after 34 days of Al treatments, rinsed with 0.5 mM CaCl_2_ for 3 min and embedded in 7% agar. The root tips were sectioned into 40 µm slices using a microtome (VT1200; Leica, Wetzlar, Germany). All samples were stained with 0.01% morin solution (Macklin, Shanghai, China) at room temperature for 30 min and thoroughly rinsed with clean water for 1 min. Fluorescence was visualized using a fluorescence microscope, with the excitation wavelength set to 580 nm to capture morin signals.

### 4.7. Determination of Al Content

Dry root system samples (approximately 0.05 g) were weighed and transferred into a crucible. The crucible was placed on an electric heating plate and heated until the sample turned black. It was then moved to a muffle furnace and ashed at 500 °C for 12 h until a white residue was obtained. After cooling, 2 mL of a 50% hydrochloric acid solution was added to dissolve the ash. The solution was transferred to a 5 mL centrifuge tube, brought to volume with deionized water, and filtered. Aluminum content was determined using inductively coupled plasma mass spectrometry (ICP-MS).

### 4.8. Quantitative Real Time-PCR Analysis

Total RNA was extracted from patchouli roots using RNA-Solv reagent (OMEGA, BioTek, Winooski, VT, USA). Following the manufacturer’s instructions, first-strand cDNA was synthesized using a reverse transcriptase kit (Promega, Madison, WI, USA). Quantitative real-time PCR (qRT-PCR) was conducted using GoTaq qPCR Master Mix (Promega, Madison, WI, USA) on an Applied Biosystems Step One Plus Real-Time PCR system (ABI, Los Angeles, CA, USA). Primers for qRT-PCR were designed using NCBI’s online Primer-BLAST (https://www.ncbi.nlm.nih.gov/tools/primer-blast/, accessed on 29 Apirl 2025) against the patchouli genome and are listed in Supplemental Table S1.

The candidate genes for qRT-PCR were selected based on a dual rationale. Firstly, they were initially identified from our internal transcriptome sequencing data as being significantly differentially expressed in response to Al treatment. Secondly, and crucially, their selection was further justified by established literature documenting their functional roles in key processes relevant to our phenotypic observations, including cell proliferation (e.g., XLG3, ACR4) [84,85], cell wall remodeling (e.g., EXPA10, XTH23) [61,62,63,64,65], and lateral root development (e.g., RAV1, AIL6) [76,77,78,79].

The relative transcription levels of the corresponding genes in patchouli were normalized using the transcription levels of the housekeeping gene, EIF4E as described previously [86]. Gene expression analysis had four biological replicates.

### 4.9. Statistical Analysis

All statistical analyses were performed using SPSS software for one-way ANOVA and Student’s *t*-test (version 21; SPSS Institute, Chicago, IL, USA).

## 5. Conclusions

**Figure 9:**
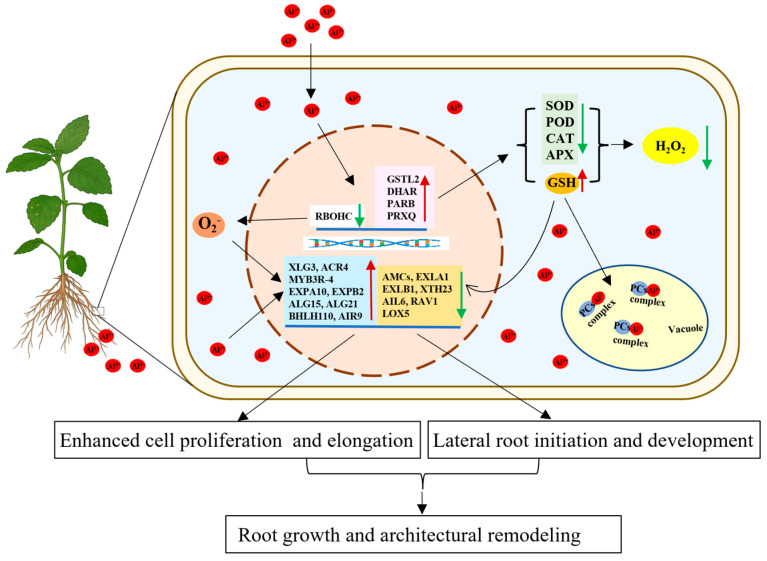
Model illustrating how aluminum promotes root growth and architectural remodeling in patchouli seedling. Al^3+^ promotes root development in patchouli through integrated mechanisms. It upregulates glutathione (GSH) biosynthesis while inhibiting enzymatic systems (e.g., SOD, POD, CAT and APX), collectively reducing H_2_O_2_ and oxidative stress. Al^3+^ also suppresses RBOHC-mediated O_2_^−^ generation, maintaining redox homeostasis. Beyond its antioxidative role, GSH may act as a signaling molecule regulating gene expression and as a chelator that facilitates Al^3+^ transport into vacuoles for detoxification. Furthermore, the observed changes in the transcript levels of genes governing cell proliferation, cell wall remodeling, and lateral root formation support the conclusion that aluminum stimulates root growth by promoting elongation, branching, and architectural adaptation.

In conclusion, we propose a model for aluminum-promoted root growth in patchouli (Figure 9). Aluminum treatment enhances glutathione (GSH) biosynthesis while concurrently suppressing key enzymatic antioxidant systems (e.g., SOD, POD, CAT, APX), collectively reducing H_2_O_2_ levels and oxidative stress. It also fine-tunes RBOHC-mediated O_2_^−^• generation to maintain redox homeostasis. Beyond its direct antioxidative role, GSH may additionally function as a signaling molecule that influences gene expression or chelates Al^3+^ for vacuolar sequestration and detoxification. Furthermore, the observed changes in the transcript levels of genes governing cell proliferation, cell wall remodeling, and lateral root formation support the conclusion that aluminum stimulates root growth by promoting elongation, branching, and architectural adaptation.

## Data Availability

Data are contained within the article and Supplementary Materials.

## Supplementary Materials

The following supporting information can be downloaded at: https://www.mdpi.com/article/10.3390/ijms262010056/s1.
