# Supplementary material for: Stimulatory Effect of Aluminum in Root Development of Pogostemon cablin: Integration of ROS Homeostasis and Gene Expression Networks
Source: Int J Mol Sci. 2025 Oct 15;26(20):10056. doi: 10.3390/ijms262010056 (PMC12564486; doi:10.3390/ijms262010056)
Supplement: Supplementary file 1 [file ijms-26-10056-s001.zip › Supplementary Figures.pdf]

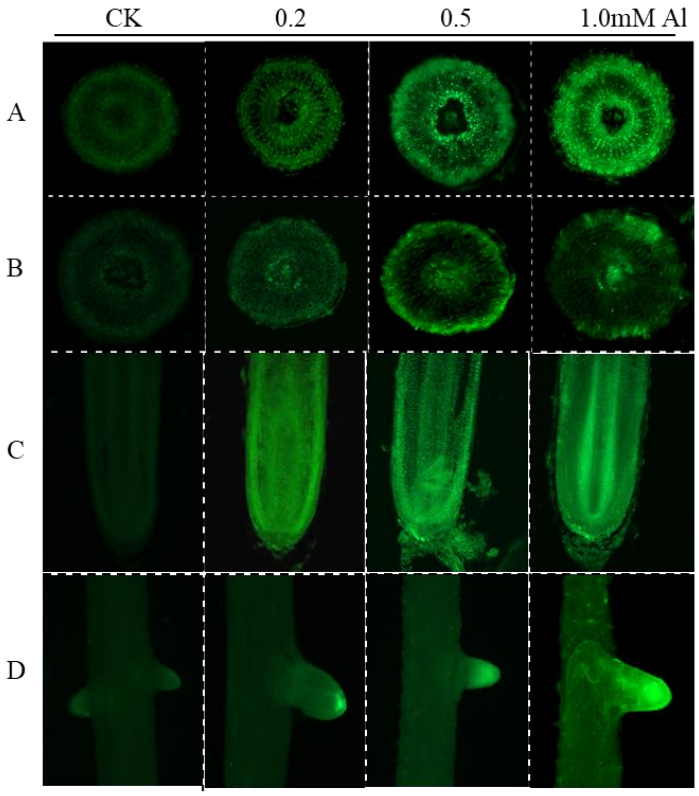


Figure S1. Localization of aluminum (Al) in roots. (A) Localization of aluminum (Al) in root meristem zone; (B) Localization of aluminum (Al) in root elongation zone; (C) Localization of Al in root tips; (D) Localization of Al in lateral root primordia. The green fluorescence is due to the morin staining of Al in the root tip cells. Bar= 100μm.

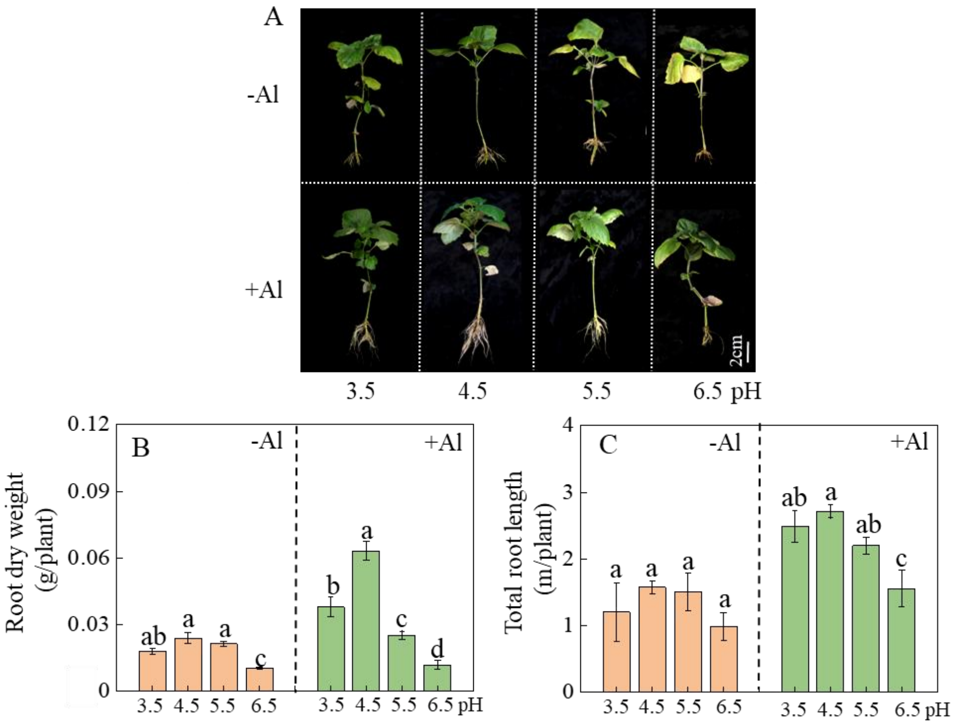


Figure S2. Effect of pH on *root* growth of patchouli seedling. A. Dynamic growth phenotype of plants under aluminum treatment at different acidic conditions; B. Root dry weight; C. Total root length. Bar=2cm. Duncan multiple comparison was adopted to analyze the significance of data differences. The columnar chart marked with different lowercase letters demonstrates significant differences between data ($p < 0.05$).

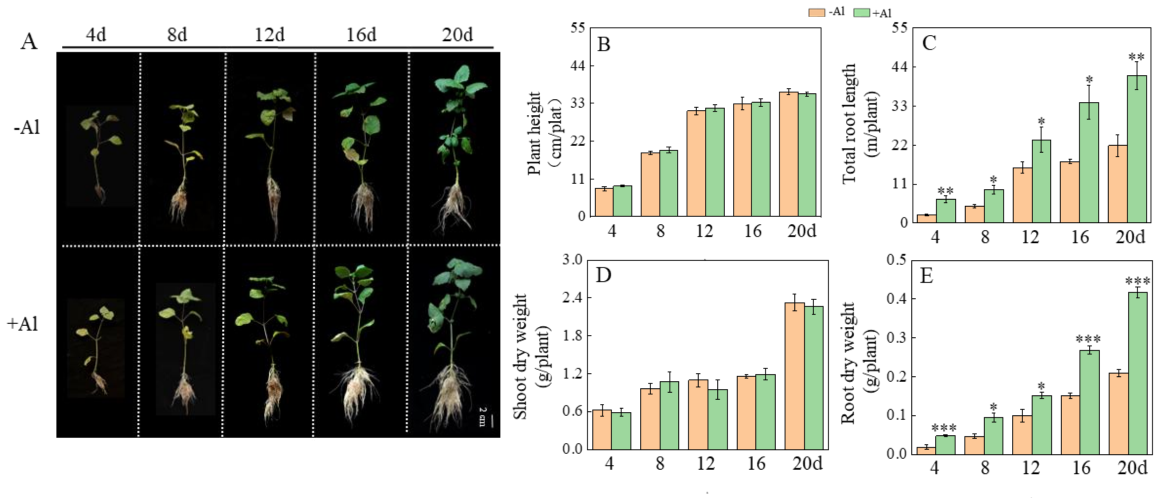


Figure S3. Effect of aluminum stress on the growth dynamics of patchouli. (A) Phenotype of seedlings treated with 0mM Al or 1mM Al for 20d; (B) Plant height; (C) Total root length; (D) shoot dry weight; (E) Root dry weight. Bar=2cm. Asterisks indicate significant differences between Al treatments and the control in the Student's t-test (*P < 0.05; **0.001 < P < 0.01; ***P < 0.001).

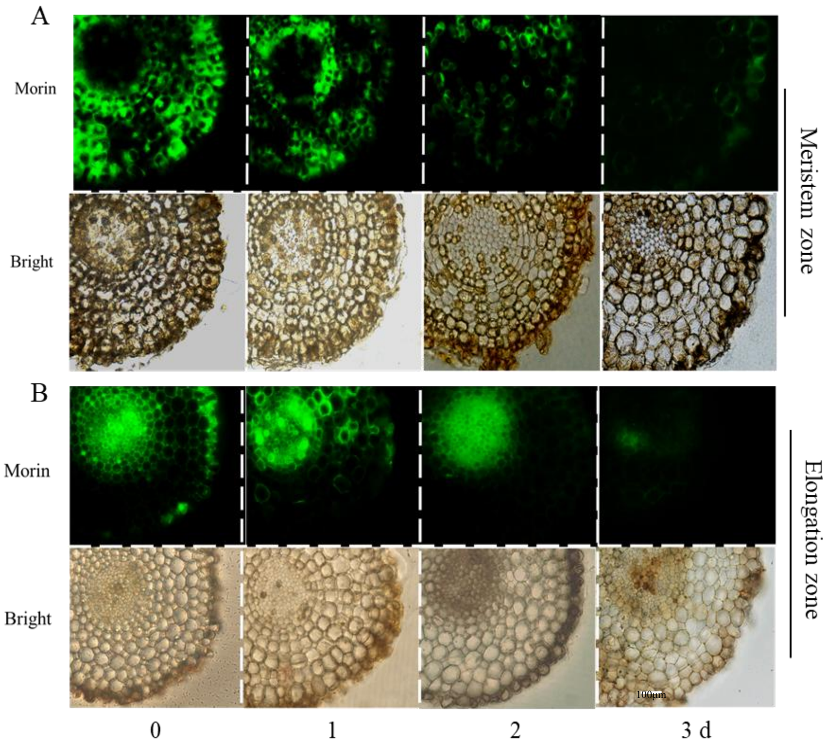


Figure S4. Localization of aluminum (Al) in root tips. (A) Localization of aluminum (Al) in root meristem zone, (B) Localization of aluminum (Al) in root elongation zone. The green fluorescence is due to the morin staining of Al in the root tip cells. Bar= 100μm.
